# Supplementary material for: Deep and fast label-free Dynamic Organellar Mapping
Source: Nat Commun. 2023 Aug 29;14:5252. doi: 10.1038/s41467-023-41000-7 (PMC10465578; doi:10.1038/s41467-023-41000-7)
Supplement: Supplementary file 1 — Supplementary Information File [file 41467_2023_41000_MOESM1_ESM.pdf]

Deep and fast label-free Dynamic Organellar Mapping

Julia P. Schessner, Vincent Albrecht, Alexandra K. Davies, Pavel Sinitcyn, Georg H.H. Borner

This file contains all supplementary figures 1-8, supplementary table 1 and supplementary note 1.

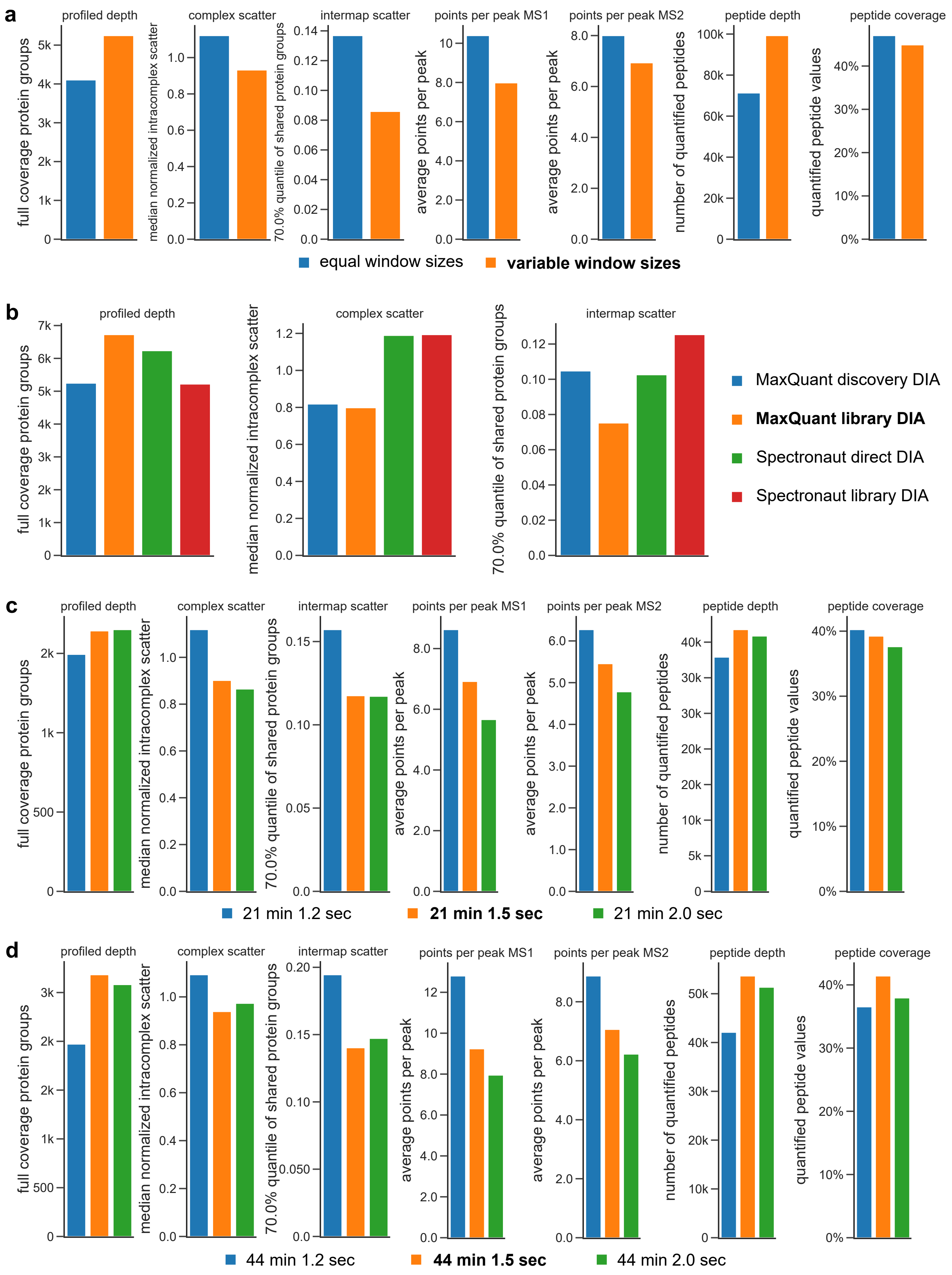

Supplementary Figure 1

Supplementary Figure 1: Optimization of the DIA workflow. Multiple datasets were acquired with different acquisition parameters and data processing strategies. The software app DOM-QC (https://domabc.bornerlab.org) was applied for unbiased performance evaluation. In each case, three replicate maps were analyzed, which all stemmed from the same subcellular fractionation experiment. We scored proteomic depth (number of protein groups profiled across all replicates), intra-complex scatter (lower scatter indicates higher quantification precision), and inter-replicate scatter (lower scatter indicates higher reproducibility). For MS method optimization additional parameters were extracted directly from the MaxQuant output files: average scans per chromatographic peak of quantified peptides at MS1 and MS2 levels, overall peptide depth (without filtering for complete profiles or replicates), peptide quantification coverage across all peptides and samples. In each panel, the configuration selected for this study is highlighted in the legend (bold, underlined). Source data are provided as a Source Data file. **A)** Window size optimization (variable DIA windows vs equally sized DIA windows). **B)** Software (MaxQuant 2.0 vs. Spectronaut 14) and database (direct/discovery vs. library) for data processing. The same set of raw files was processed in four different ways for this comparison. MaxQuant processing yields better quantification precision and reproducibility. **C)** Cycle time optimization for the 44 min gradient. The shorter the cycle time, the more quantifications per peak (q/p) were collected. However, shorter cycle times necessitate larger DIA windows, which negatively impacts on depth and precision. **D)** Cycle time optimization for the 21 min gradient.

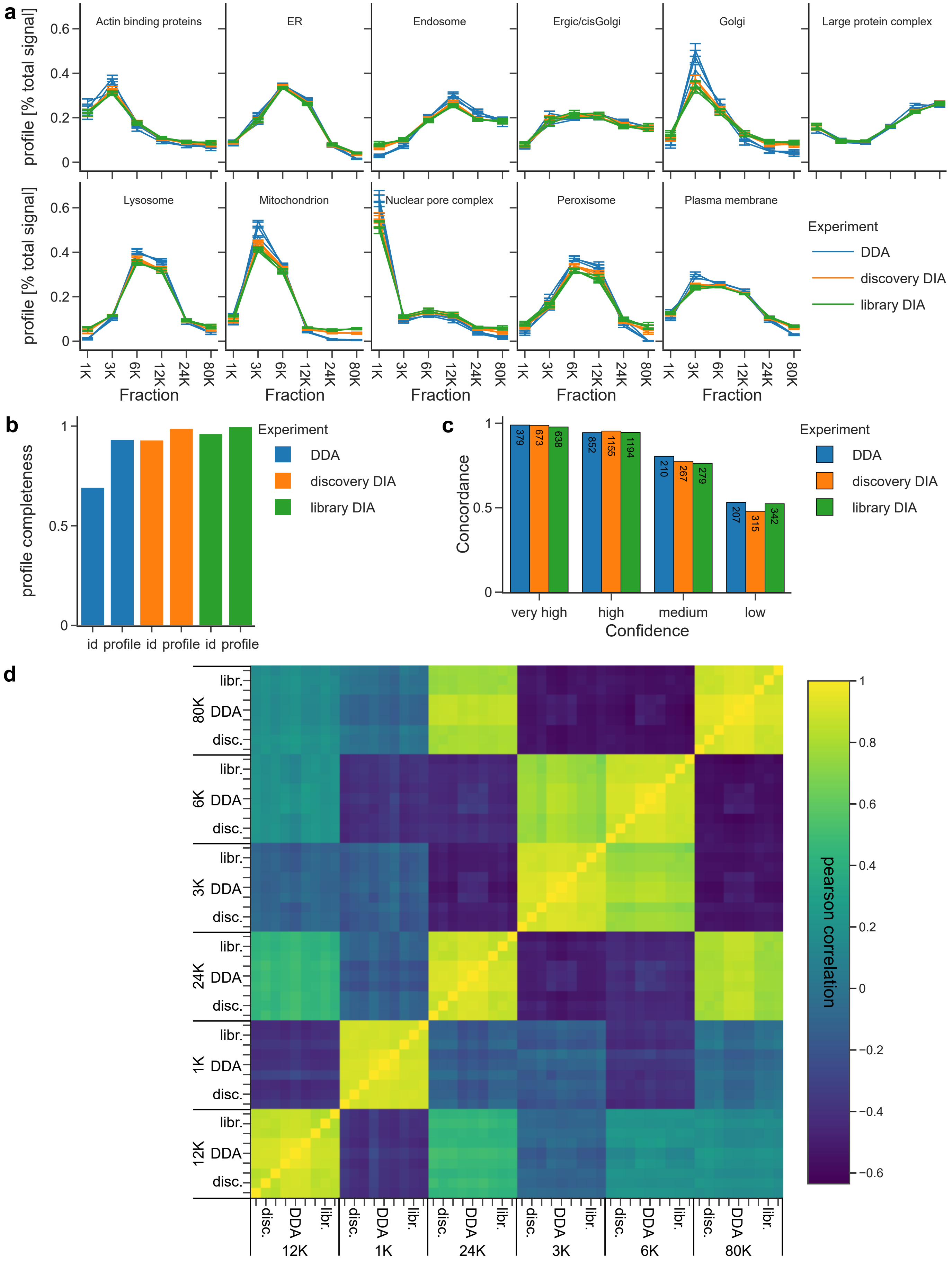

Supplementary Figure 2

Supplementary Figure 2: Comparison of DDA, discovery DIA and library DIA based maps, additional data. Source data are provided as a Source Data file. **A)** Median marker profiles of classified compartments show only minor differences in low abundant fractions, where DIA methods are more sensitive. Numbers of underlying profiles are contained in the source data file and range from 15 to 294. Error bars indicate the standard error of the mean. **B)** Profile data completeness of all identified protein groups, before and after quality filtering. **C)** Concordance of SVM predictions with previously published SILAC-based organellar maps [1,2], by prediction confidence class. Callouts indicate how many (non-marker) proteins in each class/experiment were shared with the SILAC reference maps; concordance indicates the proportion of agreement. **D)** Pearson correlation matrix between normalized profile values across all samples.

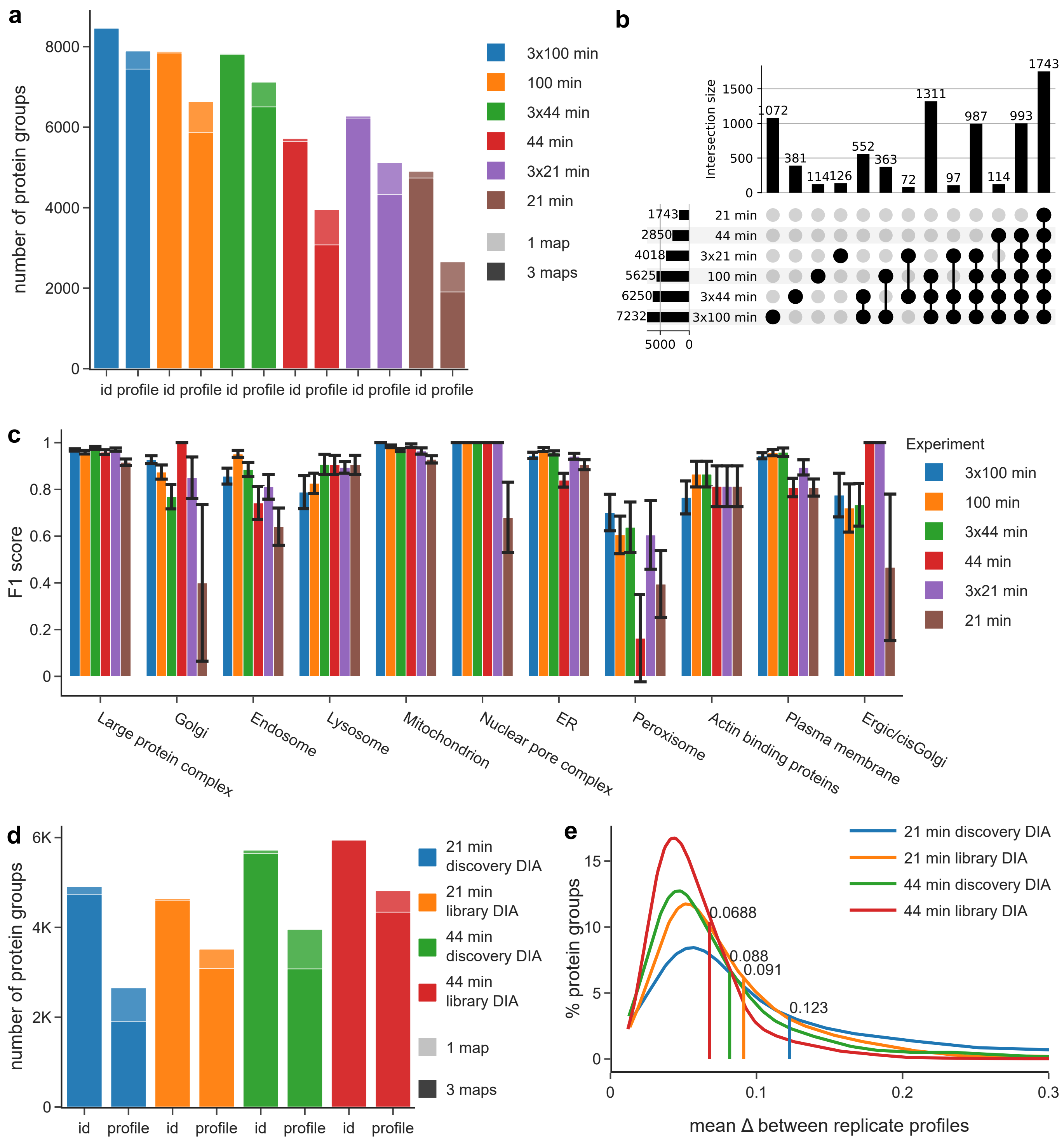

Supplementary Figure 3

Supplementary Figure 3: Comparison of DIA-DOMs performance with different LC gradients and sample fractionation, additional data. All datasets in **A-C** were generated with discovery DIA. Source data are provided as a Source Data file. **A)** Assessment of proteomic depth. id, number of protein groups identified; profile, number of protein groups with a complete abundance profile suitable for organellar mapping. Light shade, identified/profiled in at least one replicate; dark shade, identified/profiled in all three replicates. Samples were analysed either in 'single shot' with the indicated LC gradient time per subcellular fraction, or in 'triple shot' following 3 x SDB-RPS peptide fractionation. **B)** Upset-plot showing overlap of proteins profiled with different LC gradients. Combinations with intersection sizes <50 were not included. **C)** Breakdown of SVM classification performance (F1 score) by compartment and LC gradient. Mean +/- standard deviation is displayed for 20 stratified random samples of the test dataset. **D-E**, comparison of library and discovery DIA for short LC gradients. Auxiliary peptide libraries were generated by DDA, separately for each gradient (21 min gradient, ~58.7K peptides; 44 min gradient, 88.5K peptides). **D)** Proteomic depth, as in **A**. **E)** Inter-replicate scatter, for the 1,744 proteins profiled across all experiments. Lower scatter reflects higher map reproducibility.

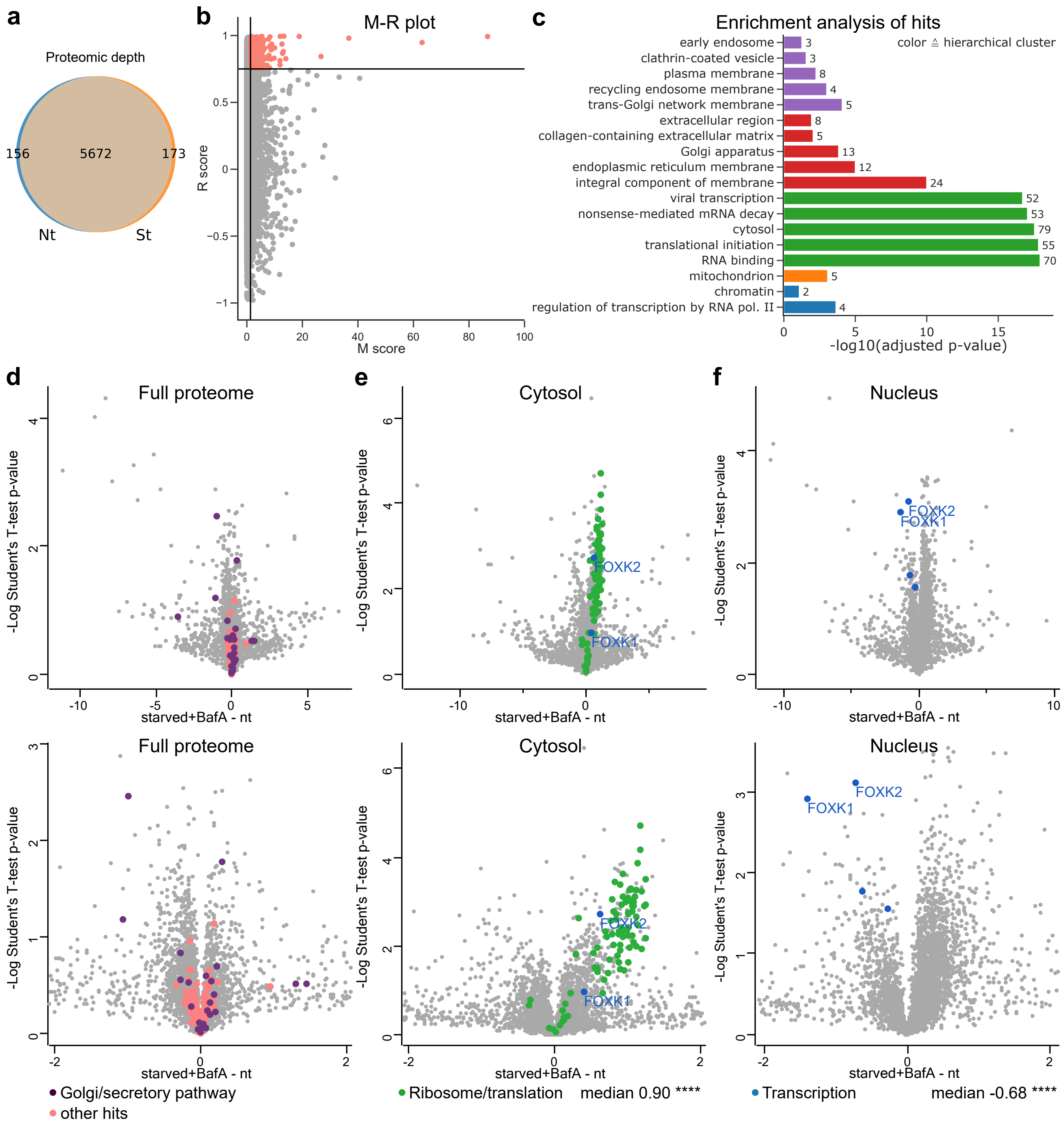

Supplementary Figure 4

Supplementary Figure 4: DIA-DOMs reveal the cellular effects of starvation and Bafilomycin A1 treatment, additional data. Source data are provided as a Source Data file. **A)** Proteomic depth and overlap of the two conditions after filtering for high quality profiles; Nt=non-treated, St=starved + 1h BafA treatment. **B)** M-R plot with full range on M and R score. **C)** Enriched GO-terms and protein families, by Fisher's exact test within all hits for the clusters shown in Fig. 4C. Terms up to a Benjamini-Hochberg corrected FDR of 10% are included. For Golgi, Endosome and Ribosome clusters, only the 5 most highly enriched terms are shown, as the others are largely redundant. The brown clusters (annotated as 'none') in Figure 4C did not show any significantly enriched terms and are thus not shown here. **D-F)** Differential abundance analysis of full proteome, nuclear fraction (1K spin pellet) and cytosol (80K spin supernatant) of HeLa cells either left untreated, or starved in the presence of BafA for 1 h**.** Triplicates (n=3) were analysed for each condition. Only proteins that were quantified at least in all replicates of one condition are included; missing values were imputed from a downshifted normal distribution. Changes were analyzed with a two-tailed Student's *t*-test. Upper panels = full range, lower panels = zoomed in on the range of 2-fold abundance changes. **D)** Full-proteome protein abundance analysis, n=5,019. Of the 164 proteins with shifted subcellular localization (as indicated by MR analysis, Fig. 4) 142 were quantified here. None of them showed a significant abundance change, and none of the clusters from Fig. 4C were significantly shifted based on a 1D annotation enrichment (not shown). **E)** Analysis of cytosolic proteins, n=4,605. 1D enrichment adjusted p-value of the ribosome/translation associated cluster is 1.4E-30. **F)** Analysis of nuclear proteins, n=5,285. 1D enrichment adjusted p-value of the 'transcription' cluster is 4.4E-3. FOKX1 and FOXK2 are reduced in the nuclear and increased in the cytosolic fraction.

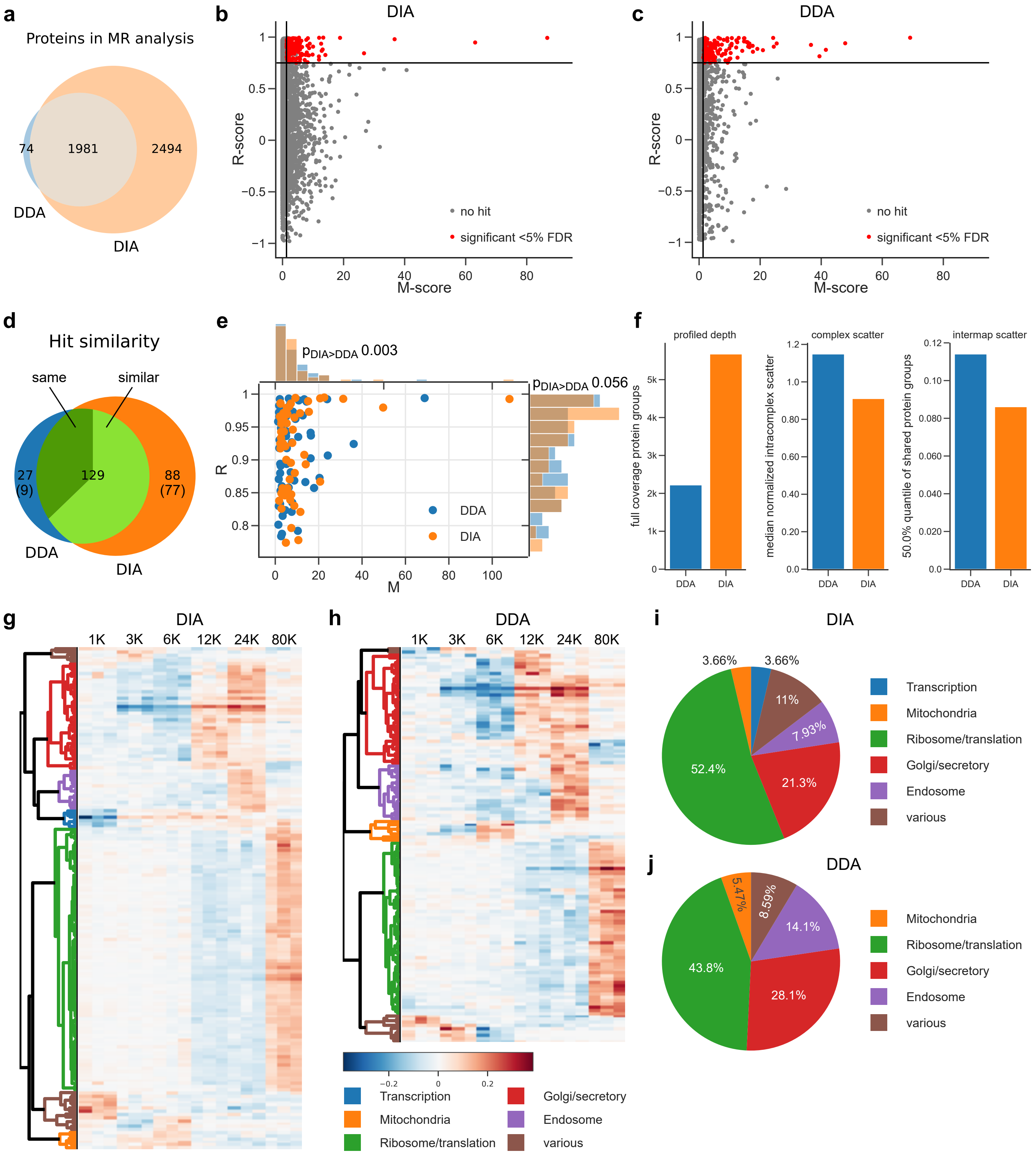

Supplementary Figure 5

Supplementary Figure 5, extending Figure 4: Comparison of DIA vs DDA DOMs for characterizing the BafA/starvation phenotype. DIA outperforms DDA across all metrics. The DIA organellar mapping experiments in Figures 4 and 5 were re-acquired with DDA, using the same HPLC and MS setup. Data were analyzed with DOM-ABC, with identical quality filters for DIA and DDA. Source data are provided as a Source Data file. **A)** Overlap of proteins used for MR analysis. DIA-DOMs achieve more than twice the profiling depth of DDA-DOMs. **B,C)** MR Plots to identify significantly shifted proteins ('hits'). DIA maps identify 164 hits, DDA maps only 128 (28% increase with DIA), at an FDR <5%, which corresponds to an M-score cut-off at 1.3. The M-score is the -log10 of the Benjamini-Hochberg corrected combination of p-values derived from three independent chi2 distributions of robust Mahalanobis distances (see Methods for details). Additionally, the R-score cut-off was 0.75, and proteins with only one replicate p-value < 0.1 were excluded. **D)** Overlap of hits. From the proteins that are common to both DIA and DDA DOMs, both methods identify similar sets of significantly shifted proteins. Numbers in brackets indicate hits that were profiled exclusively in the DIA or DDA datasets. The overlapping class includes proteins that pass high stringency filters in both DIA and DDA (N=48), as well as proteins that pass high stringency cut-offs in one condition, and more lenient cut-offs in the other condition (N=81; see methods). **E)** MR plot of the 48 high-stringency overlapping hits with marginal histograms. For a fair comparison, M-scores were calculated in the context of the 1981 proteins profiled in both DIA and DDA datasets. M and R scores have visually similar distributions with DIA and DDA; however, a paired comparison reveals that DIA results in 34 higher M- and 30 higher R-scores than DDA (p-value M scores < 0.003 , p-value R Scores < 0.056; one-sided sign tests, n = 48). DIA scores also have higher medians (Median M_{DIA} = 6.8 vs M_{DDA} = 5.8; Median R_{DIA} = 0.930 vs R_{DDA} = 0.912). These comparisons support that DIA produces better data for detecting outliers. **F)** DOM-ABC quality metrics for the DIA and DDA datasets. DIA achieves greater depth, greater quantification precision (lower intra-complex scatter), and greater reproducibility (lower replicate scatter). **G,H)** Hierarchical clustering of delta profiles of significant hits. Equivalent clusters are colour-matched. DIA and DDA yield similar clusters, but DIA identifies more proteins, and an additional cluster (blue). **I,J)** Hit assignment to categories based on hierarchical clustering (see G,H) and GO-term enrichment analysis. DIA and DDA identify functionally similar groups of hits; however, DIA identifies a further group of transcriptional regulators that is not detected by DDA. This cluster contains for example the transcription factors FOXK1 and FOXK2, which undergo a strong transition from the nucleus to the cytosol (Supplementary Figure 4 E, F).

Please note that the DIA plots in panels B and G are reproduced from Figure 4, for ease of comparison.

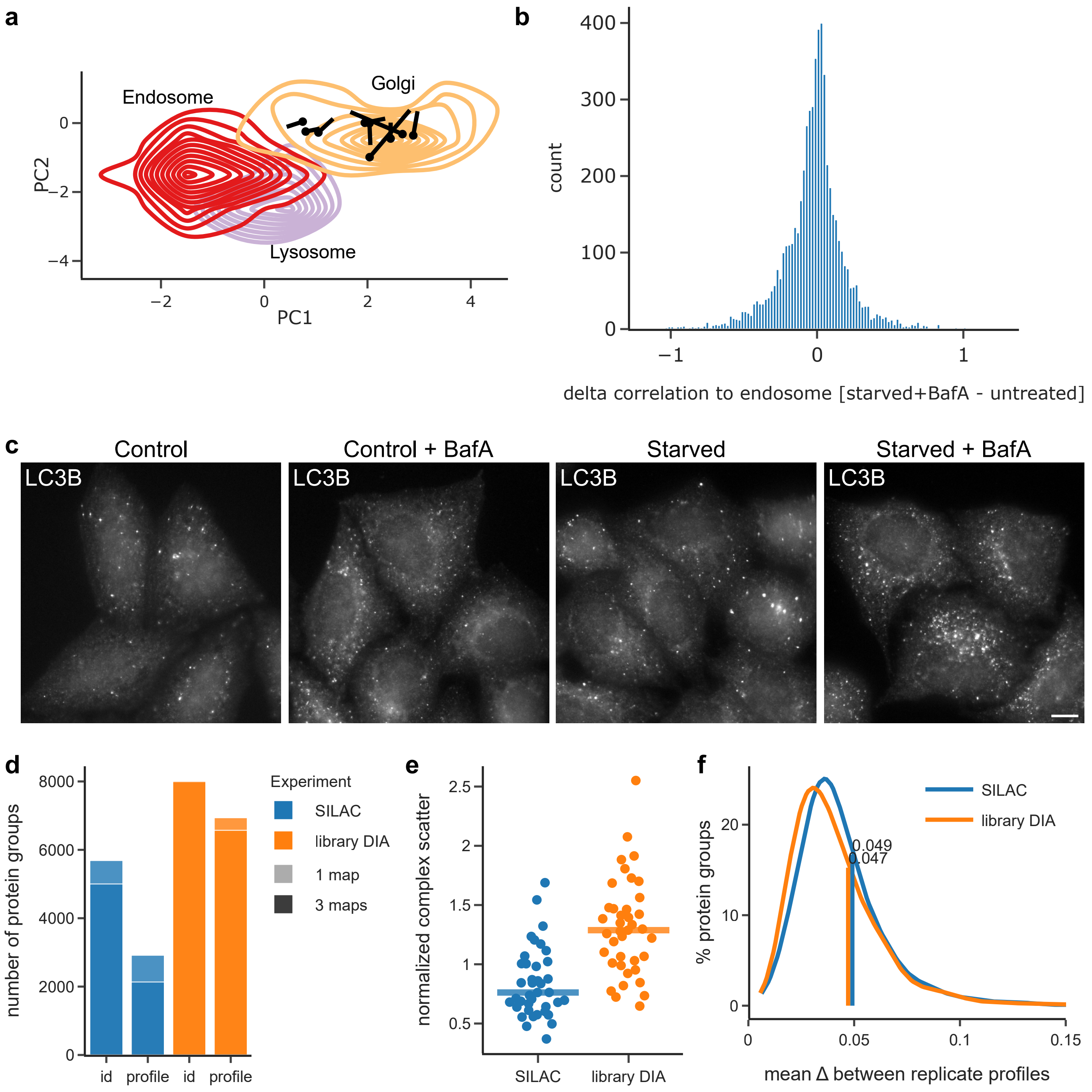

Supplementary Figure 6

Supplementary Figure 6: Individual effects of starvation vs. BafA treatment, and performance of DIA label-free vs. SILAC organellar maps. Source data are provided as a Source Data file. **A)** Localization changes of Cluster 3 Golgi proteins, similar to Figure 5C, D. **B)** Distribution of correlation changes underlying the categorization shown in Figure 5E. All protein profiles were correlated to the average profile of endosomal proteins before and after treatment, respectively. The difference between those values per protein yield a symmetrical and in the central region approximately normal distribution. See Methods for details. **C)** Widefield imaging of immunofluorescence labelling of LC3B as a marker for autophagosomes. HeLa cells were cultured for 1h in either: 1) full growth medium (Control); 2) EBSS to starve the cells (Starved); 3) full growth medium plus 100 nM BafA (Control + BafA); or 4) EBSS plus 100 nM BafA (Starved + BafA). This analysis was performed alongside the analysis of GLG1, TGOLN2 and GALNT2 shown in Fig. 6. Scale bar: 10 µm. HeLa cells have a high level of basal autophagy, so LC3B positive structures are readily detectable even under untreated control conditions. The increased number of LC3B spots in the Starved + BafA condition demonstrates that the treatment is working as expected. Brightness levels for display were set uniformly across the conditions. **D-F)** Performance of SILAC organellar maps. Organellar maps generated in a previous study [1,2] using SILAC metabolic labelling, with 100 min LC gradients and DDA, were evaluated against the reference single shot label-free DIA maps, with 100 min LC gradients, library processing, generated in this study (see Fig. 2). **D)** DIA maps have triple the profiling depth of SILAC maps (6,615 vs 2,135 protein groups after quality filtering). **E)** SILAC maps have lower intra-complex scatter than DIA maps, indicating higher quantification precision. Lines indicate the median of n=39 quantified complexes across 3 biological replicates. **F)** DIA maps have slightly better inter-replicate scatter than SILAC maps for the 2,080 proteins profiled across both experiment formats.

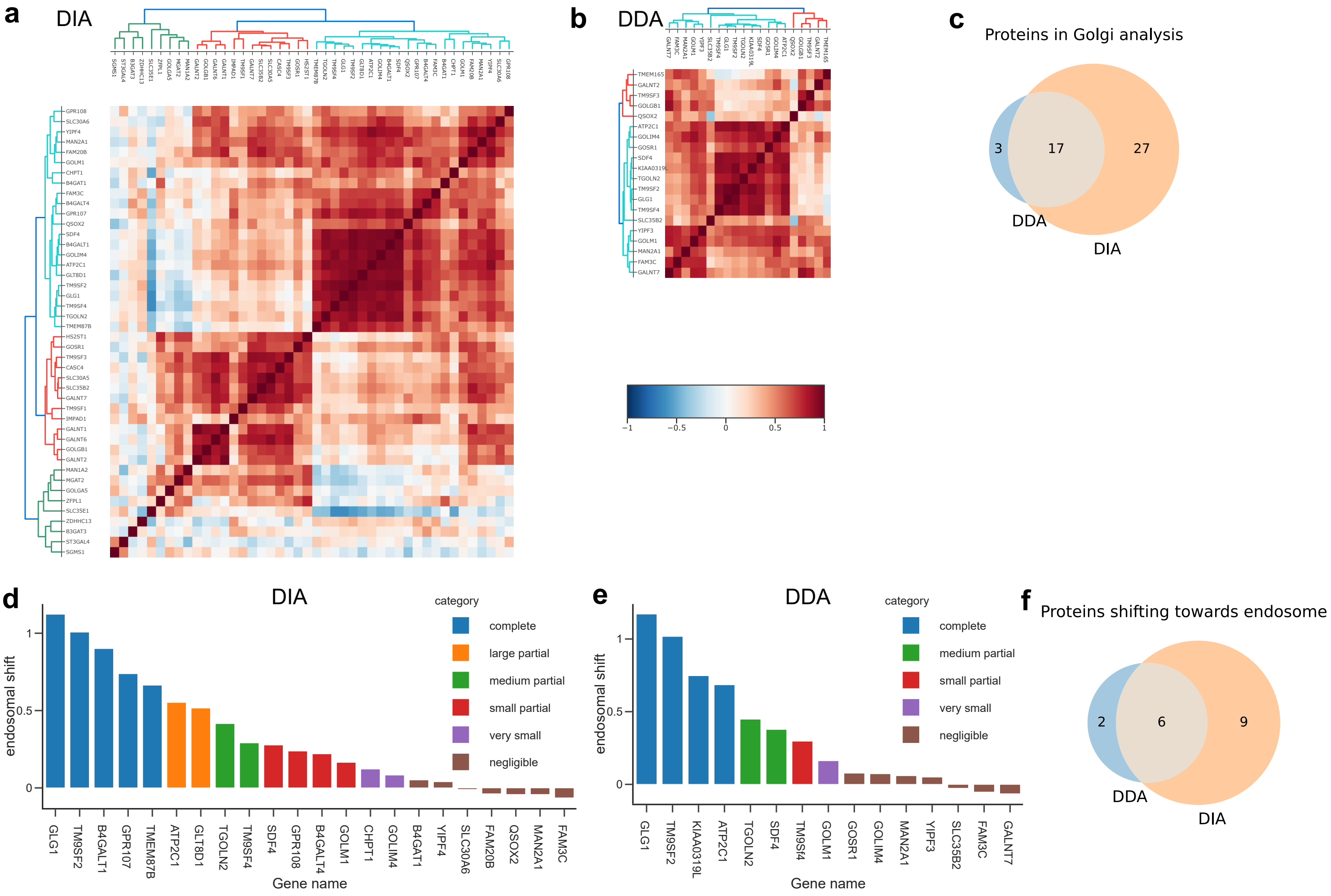

Supplementary Figure 7

Supplementary Figure 7, extending Figure 5: Comparison of DIA vs DDA DOMs for identifying cycling Golgi proteins. Source data are provided as a Source Data file. **A, B)** Hierarchical clustering of delta profiles of Golgi proteins, as in Figure 5B. DIA and DDA both identify moving and static Golgi proteins; however, DIA covers many more proteins, and identifies a third cluster not present in the DDA data. **C)** Number of proteins included in the Golgi specific clustering. DIA covers more Golgi proteins. **D, E)** Analysis of endosomal shifts in DIA and in DDA data, as in Figure 5E. **F)** Number of Golgi proteins shifting towards a more endosomal profile. DIA identifies almost twice as many cycling Golgi proteins.

Please note that the DIA plots in panels A and D are reproduced from Figure 5 for ease of comparison.

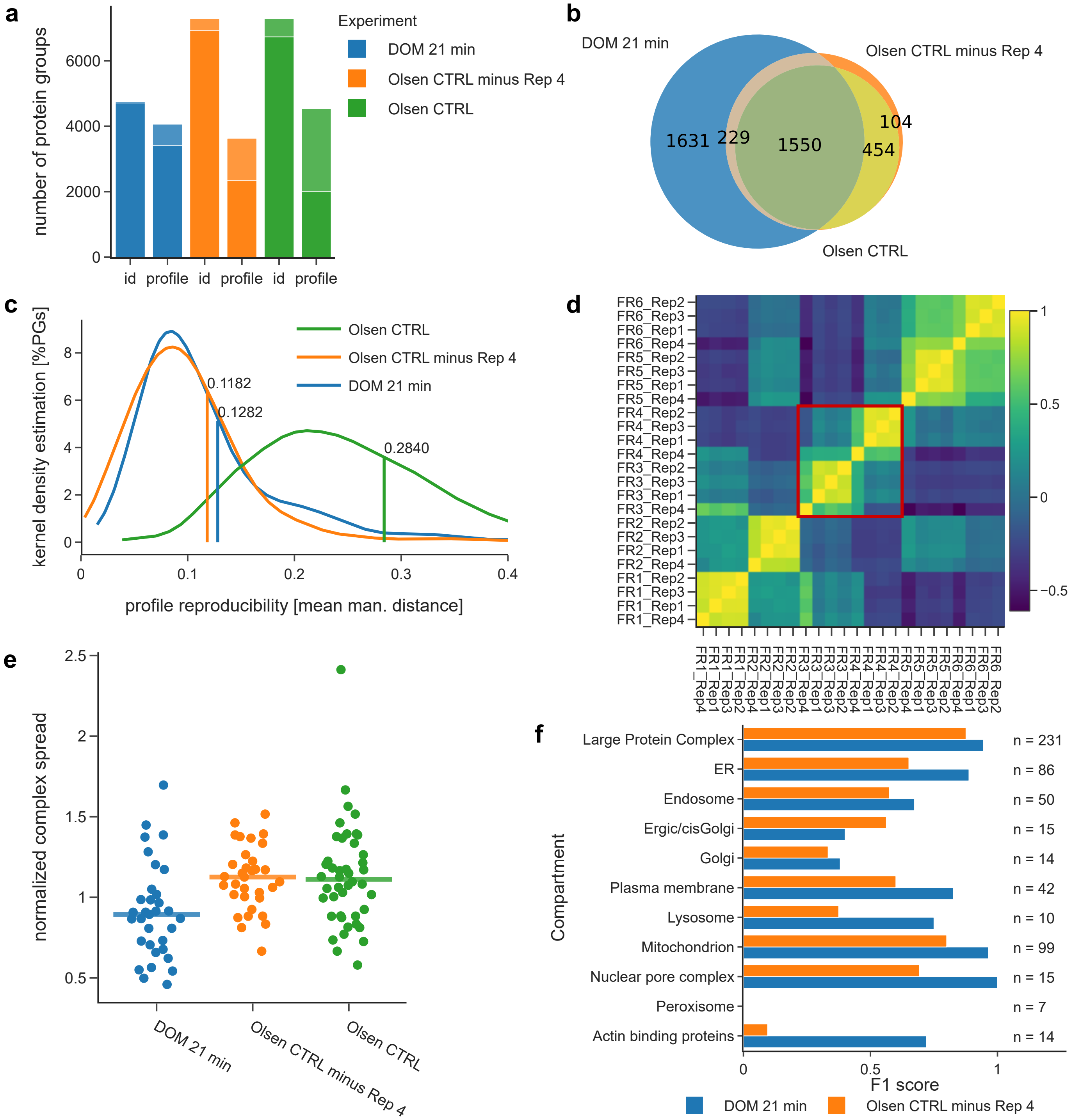

g

	Olsen	DIA-DOMs
Fractionation technique	Chemical fractionation	Differential centrifugation
Number of fractions	6	6 (7 if cytosol is included)
Minimum recommended starting material (HeLa)	1 x 15 cm dish<br>(ca. 10 million cells)	1 x 10 cm dish<br>(ca. 5 million cells)
MS run time requirements per map	6 x 21 min<br>Longer formats not tested	6 x 21 min (fast format)<br>Longer formats tested for deep mapping
Special equipment needed for fractionation	None	Benchtop ultracentrifuge
Differences in resolved compartments	Good resolution of nuclear and sub-nuclear compartments<br><br>Fair resolution of membrane bound organelles, but lumenal proteins are pooled	Good resolution of membrane-bound organelles, including lumenal proteins<br><br>Separate workflow for nuclear and cytosolic proteins

Supplementary Figure 8

Supplementary Figure 8: Comparison of DIA-DOMs and data from Martinez-Val et al. (Olsen laboratory) [3]. For data comparison we used the four untreated control replicates from the EGF-treatment of HeLa cells performed in Martinez-Val et al., and our own 21-minute gradient format. For both datasets, we only applied a consecutive value filter (n=4) using DOM-ABC and left the data unmodified otherwise. For some of the analyses, replicate 4 of the Olsen data was removed, since it was poorly correlated with the other three replicates. Source data are provided as a Source Data file. **A)** Proteomic depth of DIA-DOMs and the Olsen control maps. Dark bars represent depth across all replicates. **B)** Overlap of the protein depth covered in all replicates. **C)** Reproducibility of protein profiles. Removal of Olsen replicate 4 significantly improves the reproducibility to a comparable level as the DIA-DOMs. **D)** Pairwise correlation between all samples after profile normalization for the Olsen dataset. Replicate 4 has low correlations with the other replicates, particularly in fractions 3 and 4. **E)** Normalized scatter of stable protein complexes with at least 5 members. The median scatter remained unaffected by replicate 4 in the Olsen dataset. The median across n=33/44 quantified complexes across 3/4 biological replicates is indicated. **F)** Performance of support vector machine classification using only markers quantified in all replicates of both datasets, i.e. the three DIA-DOM maps and the three well correlated Olsen replicates (class sizes are indicated in the figure). DIA-DOMs generally perform better than the Olsen data. Due to the low number of overlapping marker proteins restricting the use of a hold-out test set, this analysis was performed using Perseus [4], as previously described [5]. **G)** Conceptual comparison of the two methods.

Supplementary Table 1: Summary of cells analysed for data shown in Figure 7, and the corresponding p-values. Reported n values correspond to number of cells analyzed over 2 independent experiments, except for the TM9SF2 6h timepoint, where cells are from 1 experiment only. For each protein, the reported p values are from a Kruskal–Wallis test with Dunn’s Post-Test, performed for comparisons to the 0h timepoint.

**Timepoint (hours)**	**0**	**0.5**	**1**	**2**	**4**	**6**	**8**
Number of cells analyzed for GLG1	184	204	168	152	197	168	188
Number of cells analyzed for TM9SF2	302	246	221	231	216	125	249
Number of cells analyzed for TGOLN2	212	158	208	173	208	200	203
Number of cells analyzed for GOLIM4	250	237	248	252	253	280	269
Number of cells analyzed for SDF4	243	172	126	136	147	170	173
p value for GLG1		2.51E-24	3.18E-54	5.18E-73	3.67E-69	1.95E-64	2.41E-77
p value for TM9SF2		1.70E-05	1.48E-65	3.72E-79	1.81E-82	3.17E-33	9.43E-93
p value for TGOLN2		7.86E-19	1.74E-38	1.64E-27	8.68E-44	2.71E-46	3.91E-37
p value for GOLIM4		1.35E-01	1.82E-17	7.09E-38	4.23E-45	1.62E-94	3.03E-121
p value for SDF4		3.19E-02	2.49E-01	9.50E-05	3.91E-11	1.46E-18	3.24E-16

Supplementary Note 1

Acronyms and concepts in spatial proteomics

DDA Data-dependent acquisition

DDA is the conventional acquisition mode for mass spectrometric protein analysis. Individual peptides are selected for sequencing, prioritized by their intensity. Although a high proportion of the selected peptides is usually identified, many low intensity peptides are never selected. This results in many 'missing values' over replicate runs.

DIA Data-independent acquisition

DIA is a relatively recent acquisition mode for mass spectrometric protein analysis. All peptides are selected for sequencing, by splitting the mass range into pre-defined acquisition windows, but the simultaneous fragmentation of multiple peptides makes the data analysis challenging. A key factor for peptide identification is the 'library' of peptides expected to be present in the sample. This can be a predicted *in silico* library, or the result of a separate DDA-based analysis. Typically, DIA results in more comprehensive peptide coverage than DDA, and hence provides greater data completeness over replicate runs.

Hierarchical clustering

Hierarchical clustering is a form of unsupervised clustering of profiling data. A given set of profiles is ordered such that profiles are grouped according to similarity. The result is a 'dendrogram' that sorts profiles into clusters. A heatmap displaying the actual profiles is commonly shown next to the dendrogram. No prior information about class memberships or other groupings is provided.

PCA Principal Component Analysis

PCA is a common tool for dimensionality reduction of multivariate data, including profiling data. It is particularly well-suited for graphically displaying multivariate data as a 2-D scatter plot. PCA derives so-called 'principal components' that preserve the information, or variability, from the original variables. Each principal component is a linear combination all original variables, with individual weights (called 'loadings'.) Every PC is non-redundant with the other PCs (i.e., uncorrelated). Usually, the first few PCs carry a large proportion of the information contained in the original dataset. Therefore, plotting the data in PC space (eg PC1 vs PC2) usually allows a 2D-visual representation of most of the information in the data, and hence the underlying data structure. In the PCA plots shown here, every scatter point represents a protein; proteins with similar localizations cluster together. PCA is unsupervised, i.e. no information about class memberships or other groupings is provided prior to the analysis.

PGs Protein Groups

A proteomics analysis results in the identification and quantification of many individual peptides. However, sometimes a set of peptides matches multiple proteins (e.g. different isoforms of the same protein, or closely related proteins), which cannot be unambiguously distinguished. They are therefore clustered into a 'protein group'. For this reason, the standard output from a shotgun proteomics experiment is not a list of identified proteins, but a list of identified 'protein groups'. Usually, many protein groups contain only one protein. Still, the use of 'PGs' is more accurate to compare the depth of a proteomic experiment than counting individual protein identifications.

Profile

In spatial proteomics, a protein's profile consists of several intensity values that reflect the protein's abundance in different subcellular fractions. Profiles may be normalized, e.g. by dividing each intensity by the sum of all intensities. The resulting 'percentage profiles' sum to 1, reflect the percentage of the protein present in each fraction, and can be compared between proteins, regardless of the proteins' absolute intensities.

SVMs Support Vector Machines

SVMs are a form of supervised machine learning for separating pre-defined classes of proteins based on profiling data. In spatial proteomics, SVMs are commonly used to predict the subcellular localizations of proteins. Typically, SVMs are first trained on a set of established compartment markers that are part of the analyzed dataset, to derive boundaries between the compartment clusters. Non-marker proteins can then be assigned to the closest marker cluster. The SVM score indicates how well a prediction fits a particular compartment cluster. Importantly, a proportion of the markers is set aside before training the SVMs. The fraction of correctly assigned markers in this 'hold-out set' is then used to estimate the predictive performance of the SVMs.

Translocation

Many proteins change subcellular localization in response to a perturbation or stimulus. These intracellular movements are termed 'translocations'. They may be complete (when the entire pool of the protein shifts), or partial; partial shifts are very common.

Commonly used metrics in spatial proteomics

F1 score

The F1 score is the harmonic mean of Recall and Precision (see below), and thus a useful overall measure of compartment prediction performance. F1 scores range from 0 (worst) to 1 (best). Unless Recall and Precision are identical, the harmonic mean is always lower than the average (arithmetic mean). Therefore, a high F1 score requires both high Precision and high Recall.

FDR False Discovery Rate

Any event that is considered statistically significant may still be the result of a chance. The FDR gauges the proportion of false positives among the number of 'significant' events: FDR = false positives / (true positives + false positives). An FDR of 5% suggests that 95% of the 'significant events' are genuine.

Intra-complex scatter / profiling precision

If two proteins have identical subcellular localizations, they should have identical profiles in a spatial proteomics experiment. This would require perfect profiling precision; any deviations reflect mass spectrometric measurement noise. However, it is not generally known *a priori* which proteins have identical localizations. To gauge profiling precision, we propose a new metric here based on stable multi-protein complexes: the intra-complex scatter. Since subunits of the same stable complex should largely have identical subcellular distributions, their profiles should be very similar. To quantify mass spectrometric measurement noise, we calculate the median profile for each complex in a curated set, and quantify the profile deviations of all complex subunits from their median profile. The result is a scatter plot as shown for example in Figure 2E. This metric is well-suited for comparing different profiling protocols. Lower scatter means that complex subunit profiles have greater similarity, and thus suggests better precision. Importantly, the method only considers proteins that are profiled across all compared sets and replicates, and complexes must have a pre-defined minimum number of subunits.

Inter-maps scatter/ profiling reproducibility

When a spatial proteomics experiment is repeated, proteins should ideally have identical profiles (if there is no biological variability). Deviations are caused by experimental noise (e.g., the subcellular fractionation), and

measurement noise from the mass spectrometry. To gauge profiling reproducibility across replicate spatial proteomics experiments, we propose a new metric here, the inter-map scatter. For each protein, we determine the median profile across all replicates. Next, we calculate the deviations of all replicate profiles from the median, and plot their distribution (see for example Figure 2F). Narrow distributions close to 0 indicate high reproducibility. If two sets of spatial proteomics experiments are compared, the plot reveals which set has better reproducibility. Importantly, the method only considers proteins that are profiled across all compared sets and replicates.

M score / Movement Score and R score / Reproducibility score

The detection of protein translocations induced by a treatment or genetic perturbation is a central challenge in spatial proteomics. We previously introduced a two-step statistical workflow, called MR analysis, to determine significant profile shifts indicative of subcellular localization changes. In a typical spatial proteomics experiment, profiling is performed before and after a treatment. For each protein, we first gauge how much its profile changes, relative to the changes of all other profiles. This is reflected in the M(ovement) score. The actual calculation of the M-Score is based on a robust multivariate outlier test, and corresponds to a -log(10) transformed corrected p-value. For example, an M score of 2 identifies a profile shift with a false discovery rate of 0.01 (1%). M scores cannot be smaller than 0; although there is no theoretical upper limit, in practice M scores are rarely >100.

Next, we determine the reproducibility of the profile shift. For each protein we calculate how well its profile shift correlates across replicates. A reproducible shift should always point in the same direction, even if the magnitude may differ between replicates – that is why correlation is a very useful measure here. R-scores range from -1 to 1. In our experience, for DOMs R scores >0.7 are indicative of a reproducible shift, but lower R-scores can also be meaningful.

Finally, we define cut-offs for both M and R scores, to identify 'significant' profile shifts.

Precision of compartment predictions

To gauge compartment prediction precision, the prediction performance for a pre-defined set of compartment markers is evaluated. These markers are called the 'hold-out set', since they are not used for training the prediction algorithm. Precision is the proportion of proteins assigned correctly to a compartment, relative to the number of all proteins assigned to that compartment (i.e. true positives / (true positives + false positives)). Precision thus ranges from 0 (worst) to 1 (best).

Proteomic depth

Proteomic depth is the total number of identified or profiled protein groups in a proteomic experiment. It is important to note that different studies quote different numbers as the achieved 'proteomic depth'. The number of identified protein groups is usually much larger than the number of protein groups with sufficient quantification coverage and quality for downstream analysis. For example, this study requires that a protein is identified in at least four consecutive subcellular fractions (out of six), with a minimum average peptide count of two per fraction, in all three map replicates, to be included in the final profiling dataset. These very stringent criteria lower the apparent proteomic depth relative to the number of identified proteins, but accurately reflect the number of proteins that can be analyzed.

Recall of compartment predictions

To gauge compartment prediction recall, the prediction performance for a pre-defined set of compartment markers is evaluated. These markers are called the 'hold-out set', since they are not used for training the prediction algorithm. Recall is the proportion of markers correctly assigned to their compartment (i.e. true positives / (true positives + false negatives)). Recall thus ranges from 0 (worst) to 1 (best). Recall can also be calculated for the entire hold-out set, across all compartments (i.e. true positives/total hold-out set size).

Supplementary References

[1] Itzhak, D.N., Tyanova, S., Cox, J., Borner, G.H.H., Global, quantitative and dynamic mapping of protein subcellular localization. *Elife* 2016, 5, e16950.

[2] Itzhak, D.N., Davies, C., Tyanova, S., Mishra, A., et al., A Mass Spectrometry-Based Approach for Mapping Protein Subcellular Localization Reveals the Spatial Proteome of Mouse Primary Neurons. *Cell Rep.* 2017, 20, 2706–2718.

[3] Martinez-Val, A., Bekker-Jensen, D.B., Steigerwald, S., Koenig, C., et al., Spatial-proteomics reveals phospho-signaling dynamics at subcellular resolution. *Nat. Commun.* 2021, 12, 7113.

[4] Tyanova, S., Temu, T., Sinitcyn, P., Carlson, A., et al., The Perseus computational platform for comprehensive analysis of (prote)omics data. *Nat. Methods* 2016, 13, 731–740.

[5] Itzhak, D.N., Schessner, J.P., Borner, G.H.H., Dynamic Organellar Maps for Spatial Proteomics. *Curr. Protoc. cell Biol.* 2019, 83, e81.
