# Supplementary material for: Deep and fast label-free Dynamic Organellar Mapping
Source: Nat Commun. 2023 Aug 29;14:5252. doi: 10.1038/s41467-023-41000-7 (PMC10465578; doi:10.1038/s41467-023-41000-7)
Supplement: Supplementary file 3 — Description of Supplementary Files [file 41467_2023_41000_MOESM3_ESM.docx]

### Filename: Supplementary Data 1

Description: Deep HeLa spatial proteome. This file contains the results of the support vector machine (SVM)-based compartment classifications for the 3 x 100 min gradient discovery DIA maps (see Fig. 3). For each protein, the SVM scores for the modelled compartments are provided.

### Filename: Supplementary Data 2

Description: This Excel sheet contains data related to Figures 4 and 5 and Supplementary figures 4 and 5. Specifically the MR data, the hierarchical clustering output and the volcano analysis are included here.

### Filename: Supplementary Data 3

### Description: Interactive database of profile shifts

This interactive Excel-table allows visualization and analysis of the protein subcellular localization shifts induced by 1h treatment with BafA1/starvation in HeLa cells.

In addition to showing M(ovement) and R(eproducibility) scores, the tool also indicates if a profile meets all criteria for a significant shift. Shifts with a False Discovery Rate (FDR) >10% or R scores <0.5 are not considered significant (Please note that these values are different from the more stringent cut-offs used in Figure 4B).

The left profile plot shows the three replicate profiles with or without treatment.

The right profile plot shows the mean profiles with or without treatment, and up to two compartment reference profiles. Please toggle reference profiles in (O6-P16) by entering ‘y’. Only the first y in each column is considered.

The tool also calculates the Pearson profile correlation of the query with each compartment reference profile. For this purpose, all three replicate profiles are combined into a single 18-datapoint profile. Control reference profiles correspond to the mean profiles of all corresponding marker proteins in the marker set (1021 proteins). Please note that prior to the calculation of reference profiles under starved conditions, marker proteins that showed significant profile shifts based on MR analysis were removed.

In columns K-M, the Query’s correlations with the two selected reference compartments (shown in the plot underneath) are displayed. Shifts in profile correlation are calculated.

In columns Q-W, this analysis is extended to all compartments. We chose this analysis over compartment assignments via SVMs, since many of the expected subcellular localization changes are partial, which is better reflected by correlation changes.

The best match of the query to a reference profile is indicated with an X. If the best match has correlation >0.9, a capital X is displayed; if it is >0.5 but <0.9, a lower case x is displayed. If it is <0.5, a question mark is displayed to indicate that there is no good match. This is typical of proteins with multiple subcellular localizations. If in addition to the top hit one or more other compartments show a correlation >0.9, they are indicated with a lower case x. This typically happens when two compartments have similar profiles (e.g., Mitochondria and Golgi).

For each compartment, the change in correlation is calculated (correl starved – correl control). Positive changes indicate a shift towards the compartment under starved conditions. The top positive change is indicated in column W.

If the top positive change corresponds to the endosomal correlation, it indicates a shift of the query protein towards endosomes. The size of the correlation shift reflects the extent of the transition (since correlations range from -1 to 1, the largest theoretically possible shift is 2; but shifts >0.5 should already be considered as relatively large).

If a query has a significant M score, a sufficiently high R score, and if its top correlation change is towards endosomes, a corresponding message will be displayed in cell J11.

**Filename: Supplementary Data 4**

Description: Misclassification matrices underlying the F1 scores displayed in Figures 2, 3 and Supplementary Figure 8.

**Filename: Supplementary Data 5**

Description: Collection of json files for reproducing or loading data analyses in DOM-ABC. Analyzed Datasets* are processed data which can be directly loaded in the benchmark section. Settings files contain all settings used for formatting and analysis of protein quantification files.
